# Micropropagation of *Alocasia longiloba* Miq and Comparative Antioxidant Properties of Ethanolic Extracts of the Field-Grown Plant, In Vitro Propagated and In Vitro-Derived Callus

**DOI:** 10.3390/plants9070816

**Published:** 2020-06-29

**Authors:** Ferid Abdulhafiz, Arifullah Mohammed, Fatimah Kayat, Suhana Zakaria, Zulhazman Hamzah, Ramachandra Reddy Pamuru, Prasada Babu Gundala, Mohd Farhan Hanif Reduan

**Affiliations:** 1 Faculty of Agro-Based Industry, University Malaysia Kelantan, Jeli 17600, Kelantan, Malaysia; ferid.f18e006f@siswa.umk.edu.my (F.A.); fatimah@umk.edu.my (F.K.); suhana@umk.edu.my (S.Z.); 2 Institute of Food Security and Sustainable Agriculture, University Malaysia Kelantan, Jeli 17600, Kelantan, Malaysia; 3 Faculty of Earth Science, University Malaysia Kelantan, Jeli 17600, Kelantan, Malaysia; zulhazman@umk.edu.my; 4 Department of Biochemistry, Yogi Vemana University, Vemanapuram, Kadapa 516003, Andhra Pradesh, India; reddyprbiotech@gmail.com; 5 Department of Microbiology, Sri Venkateswara University, Tirupathi 517502, Andhra Pradesh, India; prag.babu@gmail.com; 6 Department of Paraclinical Science, Faculty of Veterinary Medicine, University Malaysia Kelantan, Pengkalan Chepa, Kota Bharu 16100, Kelantan, Malaysia; farhan.h@umk.edu.my

**Keywords:** *Alocasia longiloba*, keladi candik, micropropagation, antioxidant, callus extract

## Abstract

In this study, an efficient micropropagation protocol was developed for *A. longiloba* and the antioxidant properties of field-grown plant, in vitro-derived greenhouse-grown plant and in vitro-derived callus extracts were compared. The *A. longiloba* seeds tested using tetrazolium chloride salt exhibited 89% viability. Due to poor germination capacity of *A. longiloba* seeds, the seeds were treated with gibberellic acid (GA_3_) or sulfuric acid (H_2_SO_4_). The maximum seed germination of 87% was observed at 30% H_2_SO_4_ treatment after 19.00 d, whereas GA_3_ treatment showed maximum germination of 53% after 22 d. In vitro shoot multiplication was carried out using various types of cytokinins alone or in combination with auxin. Among them, 6-benzyl amino purine (BAP) single treatment was found to be the best hormone. The highest shoot-length (7.26 cm) and maximum number of shoots per explant (18) were recorded at 3-mg L^−1^ BAP. For in vitro rooting, indole-3-acetic acid at 0.5-mg L^−1^ was found to be the optimum concentration. Callus was induced using various types of auxins alone or in combinations with cytokinins. The highest percentage of callus of 91 and fresh weight of 6 g was obtained with 3-mg L^−1^ IAA. The plantlets produced in the current study were subjected to acclimatization. The combination of topsoil and peat moss at 1:2 ratio was found to be the best soil media. In this study, in vitro-derived callus extract showed the highest phenolic content (538 mg GAE), followed by extracts of field-grown plant parts, i.e., fruit and petiole (504 and 300 mg GAE) while in vitro plant extract showed the lowest (98 mg GAE). Meanwhile, the highest flavonoids was recorded in petiole extract. Comparative antioxidant activity study shows, in vitro-derived callus exhibited better DPPH-radical-scavenging activity (IC_50_: 0.113-mg mL^−1^) whereas the extracts of petiole, fruit and in vitro plant showed 0.126-, 0.137- and 0.173-mg mL^−1^, respectively. At the same time, the fruit extract showed better (IC_50_: 0.088-mg mL^−1^) ABTS radical scavenging activity than all extracts tested. In conclusion, the in vitro-derived callus extract could be favored for high TPC and better DPPH scavenging activity. Hence, the present study was conducted to establish an efficient micropropagation protocol and to compare the antioxidant activity of the field-grown plant, in vitro plant and in vitro derived callus extracts of *A. longiloba*.

## 1. Introduction

Over the past decades, the use of modern biotechnological approaches such as in vitro micropropagation techniques (in vitro culture) has vastly increased due to its great potential for the propagation of high-value medicinal plants and manufacturing of high-quality natural products. In vitro culture techniques are an alternative tool for the production of medicinally important plant metabolites, rapid multiplication of endangered or rare species, production of disease free plants and plant–genome transformation [1]. These techniques assure sustainable production of plants and bioactive compounds (secondary metabolites) as they are independent of environmental factors [2,3].

Secondary metabolites are usually characterized by their structural complexity; artificial chemical synthesis is challenging or impossible [4]. Therefore, bioactive compounds are commonly obtained from natural sources. However, collection of plant material from wild populations presents risk of extinction and overexploitation. Further complications include low amounts of the bioactive compounds of interest and slow growth rate of many medicinal plants. Environmental factors such as light, temperature and CO_2_ can significantly affect the concentration of bioactive compounds in plants. All these factors render the extraction from source species very expensive and highly inefficient [5]. Therefore, there is a great need for in vitro culture for secondary metabolite production. In vitro culture techniques can eliminate the need to rely on wild plants and can alleviate pressure on wild populations [6,7]. However, the high costs of in vitro technology compared with conventional methods and the uncertainty of market demand have limited the use of in vitro techniques at commercial level [8]. Notably, however, efforts in modern biotechnological approaches (in vitro techniques) have been aimed at conserving the endangered, rare and overexploited medicinal plants that has been used for the traditional medicines throughout the world. These technologies could, therefore, have appreciable economic and environmental benefits [9,10].

In vitro techniques (callus cultures and suspension cell cultures) offer a wide range of usages in pharmaceutical sciences and herbal medicine—as well as in micropropagation for the production of plant natural products. In vitro clonal propagation of plants or tissue culture or callus can provide quality plant material that is capable of producing bioactive compounds [11,12]. Hence, in vitro techniques have become key tools and provide vast advantages over conventional methods by facilitating mass clonal propagation of homogeneous plants throughout the year, generating disease free plants and enhancements of many traits. Apart from the above-mentioned advantages, through this method, the production of valuable secondary metabolites with higher amount and better properties has become possible. These include many terpenes, alkaloids, phenolics, flavonoids, steroids and saponins and others [3,13]. The main advantage of in vitro production of secondary metabolites is that the amount of secondary metabolites produced in in vitro is greater than in parent field-grown plant parts [6,7]. In addition, it is possible to produce novel bioactive compounds that are not normally found in mother plants [14,15].

In vitro culturing of plant cells and tissues involves two major techniques, i.e., cell culture (cell suspension or callus culture) and clonal propagation techniques. In the context of cell culture, the process starts with inducing a callus for the purpose of determining best callus line and then screened for their quality, therefore the best performing lines can be used for cell suspensions. Cell suspensions or best callus line can be used for plant regeneration and extraction of secondary metabolites. While the second method of plant cell culture is based on mass propagation of plant either by using cell culture or an explant (i.e., seed, meristem, nodes, etc.) derived from potential candidate plants [16]. Micropropagation protocols has to be established for each species and plant parts as well in order to determine the amount of macro and micro mineral nutrients, vitamins, plant growth hormones, temperature, photoperiod and light intensity needs to the plant. This will allow high growth performance, survival and production of valuable natural substances [17].

Secondary metabolites can be used as a medicine, flavorings, food additives, pharmaceuticals, fragrances and agrochemicals. Several studies has been carried out for obtaining secondary metabolites for the production of pharmaceuticals, agrochemicals, cosmetics, enzyme and plant growth hormones from callus cultures as well as from in vitro propagated plants [6,18]. Secondary metabolites such as phenolic compounds are the most abundant in plant and playing major roles in plant’s defense mechanisms. The phenolic compound extracted from plants have been shown to provide many biologic activities such as anti-inflammatory, antioxidant, and anticancer [7].

It has been well reported that ROS (reactive oxygen species) are the main responsible agent for the development of various disease or disorders like cancer, diabetes, hyperuricemia, gout and inflammatory diseases [3,19]. ROS or free radicals found in the biologic systems can damage various molecules such as DNA and inhibit cell function. Many synthetic drugs have been used for their pharmacological value to treat various diseases caused by oxidative damages (ROS), however, the prolonged use of these drugs treatments has been causing serious side effects [20]. Therefore, it is necessary to identify and isolate a natural antioxidant from plant to protect oxidative damage and health disorder. The antioxidant activity of herbal extracts has been shown to be involved in the reduction of free radical formation. The DPPH and ABTS molecules which contains stable free radicals are commonly used to evaluate the free radical scavenging activity of plant extracts. Scavenging activity (anti-radical) capacity of plant extracts can be assessed by measuring decrease in the absorbance of DPPH and ABTS at various wavelengths [6].

*Alocasia longiloba* is belongs to Araceae family, which is the most morphologically diverse genera in this family [21,22]. The plant is small to usually robust, evergreen to sometimes seasonally dormant, terrestrial herbs up to 150 cm tall and rhizomatous plant. The fruit size is about 4–7 cm long, glossy green initially, but papery-marcescent as fruits reach full size, shed by rotting prior to fruit ripeness; fruits globose-ellipsoid, 1.5 × 0.75 cm, green [23,24]. *A. longiloba* is locally known as keladi candik in Kelantan, Malaysia. The traditional use had attributed a number of properties to *A. longiloba*. These include wound healing and as an anti-inflammatory [25]. The juice prepared from fruit and/or paste prepared from petiole are mostly used and externally applied onto wounded skin to relive the painful inflammation and heals wounds [26]. Das et al. [27], reported that the juice from the petiole is used against a cough and fever. Additionally, the rhizome paste is used as a poultice to treat furuncle. Our previous study showed that *A. longiloba* extracts possess various bioactive compounds which are known to have pharmacological relevance. Among the compounds identified in the *A. longiloba* extracts, Alkaloids, flavonoids and phenolic compounds are widely reported to have high medicinal values like antioxidant, anti-inflammatory and anti-gout activities [28].

Previously, micropropagation of *A. longiloba* was attempted by Bhatt et al. [22] using rhizomes as starting material, however, to the best our knowledge no research study has been conducted using seed as an explant. Here, we aimed to develop an efficient micropropagation protocol for *A. longiloba* from seed and to compare the antioxidant activity between field-grown plant parts, in vitro-derived greenhouse-grown plant and in vitro derived callus.

## 2. Materials and Methods

### 2.1. Chemicals and Reagents

All the chemicals used were analytical grade. Clorox (5.25% of sodium hypochlorite), sulfuric acid, sucrose, agar, sodium hydroxide (NaOH), hydrochloric acid (HCl), indole-3-acetic acid (IAA), picloram, α-naphthalene acetic acid (NAA), 6-Benzylaminopurine (BAP), kinetin (Kin), Naphthalene acetic acid (NAA), ethanol, distilled water, 2, 2-diphenyl-1-picrylhydrazyl (DPPH), 2,2′-Azino-bis-(3-ethylbenzothiazoline-6-sulfonic acid (ABTS), quercetin, ascorbic acid, sodium nitrate, aluminum chloride, potassium persulfate, Folin–Ciocalteu reagent and 2,3,5-triphenyl-tetrazolium chloride (TZ), were purchased from Sigma-Aldrich GmbH (Munich, Germany). Free peat (peat moss Holland) soil media were purchased from local nursery. UV-Vis spectrophotometer were used to measure absorbance (UV-Vis. Cary 4000, Agilent, UK). Rotary evaporator (Rotavapor R-200, Switzerland).

### 2.2. Plant Material and Seed Preparation

**Figure 1:**
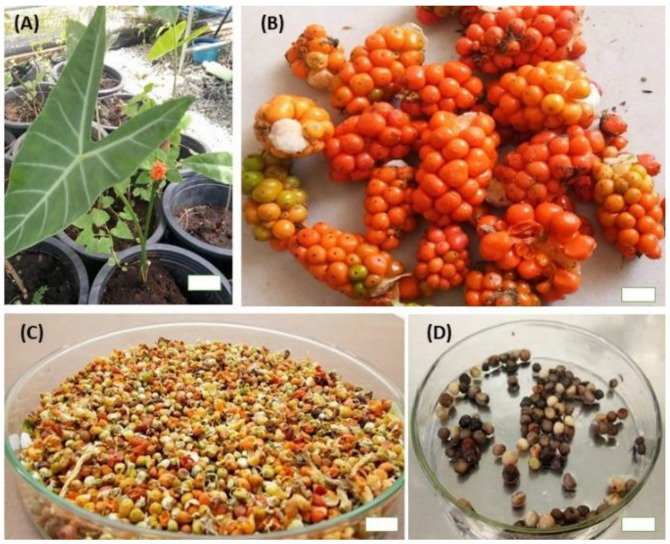
Plant material used to establish micro-propagation of *A. longiloba*. (**A**) *A. longiloba* plant collected from field; (**B**) ripened fruit harvested from field grown *A. longiloba* plant; (**C**) dried fruit; (**D**) seeds isolated from dried fruit of *A. longiloba* (bar: 1 cm).

The mature fruits of *A. longiloba* (Figure 1B) were collected from Kota Bahru, Kelantan, Malaysia (6.1211° N, 102.3178° E) between January and February 2019. The fruit were washed under running water followed by rinsing in sterile distilled water to remove the dusts completely. The seed were then separated carefully by hand and dried in room temperature for a two-week.

## 3. In Vitro Propagation of *A. longiloba*

### 3.1. Seed Viability Test

The viability of the seeds was tested using 2,3,5-triphenyl-tetrazolium chloride salt (TZ) following the methodology adopted by Da Silva et al. [29] with some modification. The test was carried out on 100 seeds. Briefly, the seeds were imbibed in sterile distilled water for 18 h at 25 ± 2 °C to soften the tegument and activate the enzyme systems. Then the seeds were immersed in 1% tetrazolium solution (pH 7.0 ± 2) and incubated at 45 °C for 6 h in the dark condition. At the end of the incubation period, the seeds were washed with sterile distilled water and examined individually under stereo microscope (M-45 series zoom stereo microscope, USA). Changes in tissue color were examined and viable and non-viable seed classes were established based on bright red color stain presence in each embryo. The percentage of seed viability was calculated as the number of stained embryos/total no. of embryos multiplied by 100.

### 3.2. Seed Surface Sterilization

The dried seed of *A. longiloba* were washed thoroughly under running tap water for 30 min followed by surface sterilization with different concentration (10%, 20%, 30% and 40%) of Clorox (sodium hypochlorite 5.25%) with a few drops of tween-20 (Sigma-Aldrich, St. Louis, MO, USA). The seeds were then shaken on an orbital shaker at 80 rpm for 20 min. The sterilized seeds then were washed with sterile distilled water to remove a trace of Clorox and then blot dried on sterilized Whatman filter study (90 mm). Seeds were subsequently cultured on a medium consisting of MS medium [30], supplemented with 30 g L^−1^ sucrose, 2.78 g L^−1^ Gelrite without plant growth regulator. The pH was adjusted to 5.7 ± 0.2 prior to adding agar and then autoclaved at 121 °C at 103 kPa for 20 min. The cultures were incubated at 25 ± 2 °C with a 16-h photoperiod provided by cool white fluorescent lights. The cultures were observed for four weeks. Two parameters were recorded such as contamination percentage and percentage of explant survival.

### 3.3. Pre-germination Treatments with Sulfuric Acid and GA_3_

Based on our preliminary study on seed germination without pre-germination treatments, the seed germination period of *Alocasia longiloba* seeds were found to be longer that took about 4–8 weeks. Therefore, two seed germination experiments were conducted in order to develop a reliable protocol (pre-germination protocol) for breaking seed dormancy and maximize germination of *A. longiloba* seeds. In the first experiment the seeds were scarified with different concentration of sulfuric acid (10%, 20%, 30% and 40%) for 15 min and then rinsed in distilled water for 10 min to remove the trace of acids solution. Then, surface sterilization was carried out with 40% Clorox for 20 min and then washed with sterile distilled water three times each time 1 min. Seeds were subsequently cultured in vitro on a medium consisting of MS basal medium supplemented with 3% sucrose and 2.78 g L^−1^ Gelrite without plant growth regulator.

In the second experiment the seeds were soaked in different concentration (5, 10, 15 and 20-mg L^−1^) of gibberellic acid (GA_3_) for 24 h. While the control seeds were soaked in sterile distilled water. The seeds then surface sterilized with 40% Clorox for 20 min and then washed with sterile distilled water three times each time 1 min. Seeds were cultured in vitro on a medium consisting of MS basal medium without plant growth regulator. Six replicates for each treatment were made with 30 seeds each one. The cultures from both experiments were observed for six weeks. Two parameters were recorded such as germination percentage and number of days taken for germination. Germination percentage calculated as the number of seeds germinated/total number seeds multiplied by 100. The culture in both experiments maintained in a growth chamber with fluorescent light (16/8 h light and dark condition.

### 3.4. Shoot Induction and Multiplication

Four weeks old in vitro raised seedlings (Figure 1D) were used for shoot induction. The cotyledon, hypocotyl and the root were removed carefully and then about 2–3 cm shoot tip were inoculated into MS medium supplemented with different concentrations (0, 1, 2, 3, 4 and 5-mg L^−1^) of cytokinins (BAP, KIN, TDZ or BAP/IAA) separately in 350 mL glass jar to study their ability for shoot induction and multiplication. Two explants were inoculated into each glass jar and six or twelve glass jars were used for each treatment. The number of multiple shoots formed, shoot height, number of roots and root length produced by each explant was recorded after four weeks of culturing. To get enough explants for further experiment, the aseptic in vitro plants were subcultured every four weeks on culture medium which was optimized.

### 3.5. Callus Induction from Seed Explant

The seeds of *A. longiloba* were used for callus induction study. The seed were treated with 30% sulfuric acid for 15 min followed by surface sterilization with 40% Clorox for 20 min. The explant were then washed with sterile distilled water to remove the trace of Clorox. The disinfected seeds were inoculated aseptically in a jar containing approximately 25 mL medium for callus induction. MS medium supplemented with various concentrations (0, 1, 1.5, 2, 2.5 and 3-mg L^−1^) of auxins (IAA and picloram) were tested for callus induction. The culture were incubated at 25 ± 2 °C under cool fluorescent light with 16/8 h light/dark cycle. The results were observed after four weeks of culture. The optimum concentration of auxin was kept as standard and tried along with different concentrations of cytokinins (BAP). Two parameters were recorded such as callus induction percentage and callus weight.

### 3.6. Root Induction and Acclimatization of Micro-Propagated Plants

To determine the effect of auxin and activated charcoal on the efficiency of in vitro rooting, the isolated shoots (2–3 cm) from multiple shoot culture were cultured into glass jars containing 20 mL of MS medium supplemented with different concentrations (0.5, 1, 1.5 and 2-mg L^−1^) of IAA or 1, 1.5, 2 and 3 g L^−1^ activated charcoal supplemented with 30 g L^−1^ sucrose and 8 g L^−1^ agar under 16/8 h light/dark photoperiod. The percentage of rooting, mean number of roots per explant and root length were determined at the fourth weeks. After 4-week, well grown rooted plantlets (8–9 cm in height) were carefully taken out from culture vessels and washed thoroughly with running tap water and the root were rinsed with water to remove agar medium. Plantlets were then kept in culture room with a temperature of 25 ± 2 °C for 5 days before being transferred to the greenhouse. After five days the plantlets were planted into small plastic pot size 20 × 15 cm containing different scheme of growth medium (T1: topsoil, T2: peat moss, T3: topsoil/peat moss (1:1), T4: topsoil/peat moss (1:2), T5: topsoil/peat moss (1:3), T6: peat moss/sand (1:1)). The plant was then kept in the green house with 100% shade. Percentage of plantlets survived, number of leaves, leaves size and plant height was determined after 45 days.

### 3.7. Preparation of Extracts and Quantitative Phytochemical Study

#### 3.7.1. Preparation of Extracts

The field-grown plant parts, i.e., fruit and petiole and tissue culture raised plant and in vitro-derived callus were used to study the comparative quantitative phytochemical and antioxidant activity. The fruit and petiole parts were dried in the oven at 40 °C for 72 h and then grinded to a fine powder using electrical blender. The dried powder was extracted using Soxhlet extractor, briefly a 30 g of powder were extracted in 300 mL ethanol for six rounds and the filtrate was concentrated using evaporator at 45 °C. The extracts then stored at 4 °C in a storage vial until further experiment. The callus extracts were prepared the same way using friable callus induced in vitro using various types of plant growth regulators.

#### 3.7.2. Determination of Total Phenolic Content

The total phenolics content of ethanolic extracts of field-grown plant parts, i.e., petiole and fruit, in vitro-derived greenhouse-grown plant and in vitro derived callus were determined using Folin-Ciocalteu reagent (FC) following the procedure by [31] with minor modification. Briefly, 100 μL of different concentrations of test sample was mixed with 1 mL of diluted 2-N FC reagent (1:10). After 10 min, 1 mL of 7.5% (*w/v*) sodium carbonate solution was added to the mixture and incubated in the dark for 90 min. The absorbance was recorded at 725 nm. The phenolic content was calculated from calibration curve and expressed as milligrams of gallic acid equivalents per gram of dry weight (mg GAE / g extract).

#### 3.7.3. Determination of Total Flavonoids Content

The total flavonoid content was determined by the aluminum chloride colorimetric method following the procedure by Jing with a minor modification [32]. Briefly, 50 μL of 5% (*w/v*) sodium nitrate solution was added to 0.5 mL of various concentrations of extract and then it was allowed to react for 5 min. Then, 50 μL of 10% (*w/v*) aluminum chloride solution was added. After 5 min, 250 μL of 4% (*w/v*) NaOH were added into the mixture. The absorbance was measured at 518 nm immediately. Quercetin was used to make standard calibration curve. The concentration of total flavonoid was expressed as milligrams of quercetin equivalents per g of dry extract (mg QCE/g extract).

### 3.8. Antioxidant Activity

#### 3.8.1. 1-Diphenyl-2-picryl-hydrazyl Assay (DPPH) Assay

The ability of plant extract to scavenge the DPPH free radicals was determined following the method of [33] with minor modifications. Briefly, 2 mL of test sample (12.5, 25, 50, 100, 200 and 400 μg mL^−1^) were mixed with 2 mL 0.004% *w/v* DPPH solution dissolved in MeOH and then incubated in the dark for 30 min. The absorbance was recorded at 517 nm. The capacity of the extracts to scavenge free radicals was calculated using Equation (1):

DPPH scavenging effect (%) = (A1 − A0/A1) × 100(1)

where A1 was the absorbance of control (DPPH solution only); A0 was the absorbance of test extract at various concentrations with DPPH.

#### 3.8.2. ABTS Free Radical Scavenging Assay

The antioxidant activity of test sample to scavenge the ABTS radicals was determined following the method of [34] with some modifications. ABTS solution was prepared by mixing equal volumes of 7 mM ABTS with 2.45 mM potassium persulfate. Then mixture allowed to stand in the dark at room temperature for 16 h. This solution was suitably diluted with methanol to yield an absorbance of 0.701 ± 0.03 at 734 nm and then used for anti-oxidant assay. Briefly, 1 mL of different concentrations of extract (12.5, 25, 50, 100, 200 and 400 μg mL−1) were added to 2 mL of to the above activated pre-generated ABTS solution and the vortexed for 1 min. After 10 min incubation in the dark, then the absorbance was measure at 734 nm, using methanol as a blank. The result was compared with control (only ABTS solution) having absorbance 0.70. ABTS radicals scavenging activity was calculated using the formula (Equation (2)).

ABTS scavenging effect (%) = (A1 − A0/A1) × 100(2)

where A1 was the absorbance of control (ABTS solution only); A0 was the absorbance of test extract at various concentrations with ABTS.

### 3.9. Statistical Analysis

The data were analyzed by one-way ANOVA to identify significant differences among the treatment/control groups (*p* < 0.05) and means were compared by Duncan multiple range test (DMRT) using SPSS v. 21.0 statistical software program. IC_50_ were calculated through linear regression analysis. Letters (i.e., a, b, c, d, e) were used to indicate statistical differences between means. All data were presented as mean ± standard error.

## 4. Results and Discussion

### 4.1. In Vitro Propagation of A. longiloba

#### 4.1.1. Seed Viability Test

**Figure 2:**
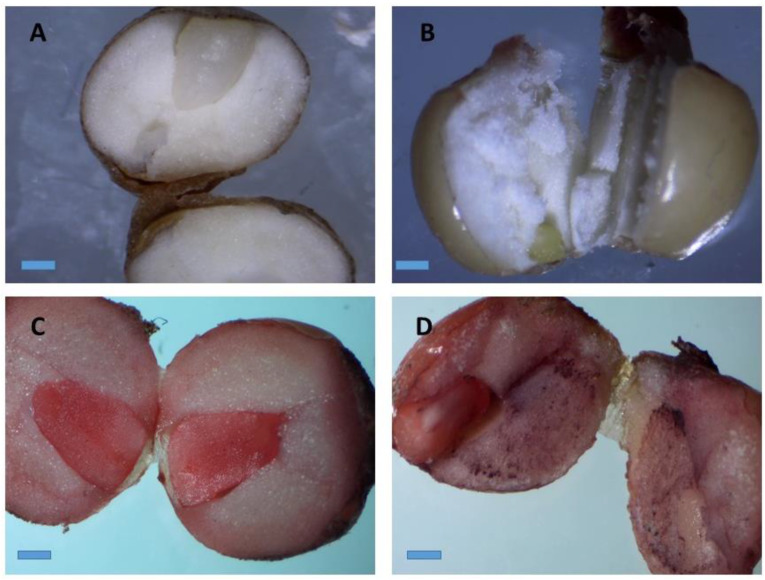
Stereo microscopic images of stained *A. longiloba* embryos by immersion in a tetrazolium solution. (**A**) Natural color of *A. longiloba* seed; (**B**) non-viable seed which retained its original color after TZ treatment; (**C**) and (**D**) red–pink color stained on the embryo after TZ treatment which indicates that the seed is viable (magnification: 40×; bar: 1 cm).

The tetrazolium test carried out with 100 seeds exhibited the seeds viability of 89%. The viable seed had developed red pink color on their embryo whereas non-viable seed had retained their original color (Figure 2B). The TZ test is the most reliable method used to estimate the seed viability and quality. The TZ solution reacts with the living cells and produces a colored salt called formazan, which will enable to examine the viable and non-viable seed. Moreover, this test allows a rapid and definitive evaluation of physiological quality of the seeds [35]. Hence, evaluation of viability of the seeds may help in making decision on choice of seeds. To the best of our knowledge no report has been made on seed viability and micro-propagation of *A. longiloba* using seed as an explant. Therefore, this study reports for the first time the viability and the pretreatment protocol to determine the physiological quality of *A. longiloba* seeds.

#### 4.1.2. Effect of Different Concentration of Clorox on the Level of Microbial Contamination

**Figure 3:**
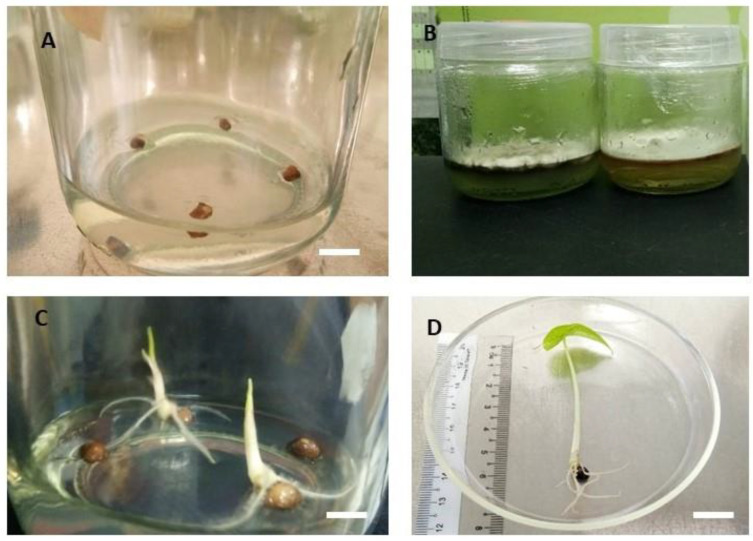
Seed culture of *A. longiloba*. (**A**) seed on the first day before contamination sets in; (**B**) contaminated culture; (**C**) germinated seed after 2–3 weeks of culture (bar = 1 cm); (**D**) four weeks old germinated seed explant (bar = 1 cm).

Different percentage of decontamination were observed among the cultures with respect to different concentration of Clorox treatments. The contamination percentage was calculated at the end of four weeks of culturing. The highest contamination percentage (62%) was observed at T1 cultures in which explants were sterilized with 10% Clorox, followed by T2 (20%) which had recorded 37% microbial contamination (Table 1). Meanwhile, the lowest contamination percentage were recorded at Clorox treatment T4 (40%) which were recorded only 5.8% microbial contamination (Figure 3C). Concurrently, the highest mean seed survival percentage (79%) were from the sterilization treatment of T3 (30%), followed by T4 (40%), these two treatments did not differ significantly from each other. The result is in agreement with the report by [36], who developed an efficient sterilization condition for the initiation of *Ipomoea batatas* seed culture.

In this experiment, it was witnessed that as the concentration of the sterilant increased, the microbial contamination had reduced significantly. In addition [36] reported that increase in the concentration could reduce microbial contaminants, but it may lead to death of the culture materials. This was evident in the current result when the concentration of Clorox increased. Sodium hypochlorite (NaOCl), also commercially known as Clorox, is widely used as a surface sterilizing agent in the prevention and treatment of microbial contamination in the plant cell and tissue culture experiments. The active components in Clorox are water and sodium hypochlorite that can used as disinfectant. [37], reported that sodium hypochlorite could act as an oxidizing agent that kills a large range of pathogens and has the potential as a germicide agent. Therefore, the current experiment demonstrates that Clorox treatment at the concentration of 40% for 15 min can be used as a decontaminant agent for seed culture of *A. longiloba*.

#### 4.1.3. Effect of Sulfuric Acid and Gibberellic Acid Treatment on Seed Germination

Sulfuric acid treatment increased seed germination as compared with the control. The increase was directly proportional to the increase in sulfuric acid concentrations. The highest germination percentage (87.50% ± 5.59%) was observed on seeds treated with 30% sulfuric acid after 19.00 ± 1.15 d, followed by (79.16% ± 7.68%) germination percentage on seeds treated with 20% sulfuric acid after 20.50 ± 3.06 d (Table 2). The minimum seed germination percentage (25.00% ± 6.45%) was observed in control treatment that took 30.16 ± 1.57 d to commence germination. All concentration of sulfuric acid reduced the number of days to seed germination in the range of between 17–37% and increased the germination percentage by 80% to 250%. In the current study, sulfuric acid treatment at 30% was the optimum concentration and was found to be sufficient to release seed dormancy and speeding up the seed germination process.

The Gibberellic acid treatment increased seed germination as compared with the control group. The highest percentage (53.33% ± 9.88%) of seed germination occurred when seeds were soaked with 20-mg L^−1^ GA_3_ after 22.83 ± 3.77 d. However, lower concentrations (5, 10 and 15-mg L^−1^) of GA_3_ were not found effective for increasing seed germination and significance difference with control treatment.

In the current study, we have demonstrated that sulfuric acid and GA_3_ treatments increase the seed germination and the speed of germination for *A. longiloba*. Similar increase in seed germination were reported when seed treated at similar concentrations for a wide variety of seeds such as *Trichocereus terscheckii* [38] and *Prunus avium* [39]. Moreover, the current result is in accordance with Dilaver et al. 2017, they noted that sulfuric acid treatment at 30% had significantly stimulated the seed germination percentage of *Astragalus vulnerariae* plant. According to [40], the presence of hard seed coat, poor germinability and dormancy are the principal causes which limit seed germination of many species including *A. longiloba*. Reserve tissues wrapped around the embryo and mucilage on the outer surface of the seed coat are other factors which hinder the permeability of water and oxygen through hilum to embryos. Therefore, chemical scarification (acid treatment) could be an alternative means to overcome the problem and achieve higher germination rate. Several researchers have confirmed that, the use of sulfuric acid at concentrations (20–40%) could make a seed coat highly permeable to water and oxygen that reduces the seed germination period. Whereas lower concentration at 5–10% sulfuric acid scarification poorly affected impermeability of hilum and seed coat. Likewise, [40] reported that 30–40% acid treatment had increased the permeability of water and oxygen to embryo and activate the metabolism after contacting embryos. This could result in easy cracking of seed coat followed by emergency of radicle and stimulates the seedlings growth in short period of time in agreement with [41,42]. Therefore, the current experiment demonstrates that 30% sulfuric acid concentration could be used for in vitro seed germination of *A. longiloba* in order to boost the seed germination and stimulates seedling growth. Moreover, the seed germination protocol developed in this study can be used for healthy seed multiplication in the green house/nurseries under natural conditions.

#### 4.1.4. Effect of Different Type and Concentration of Cytokinins on Multiple Shoots Induction

**Figure 4:**
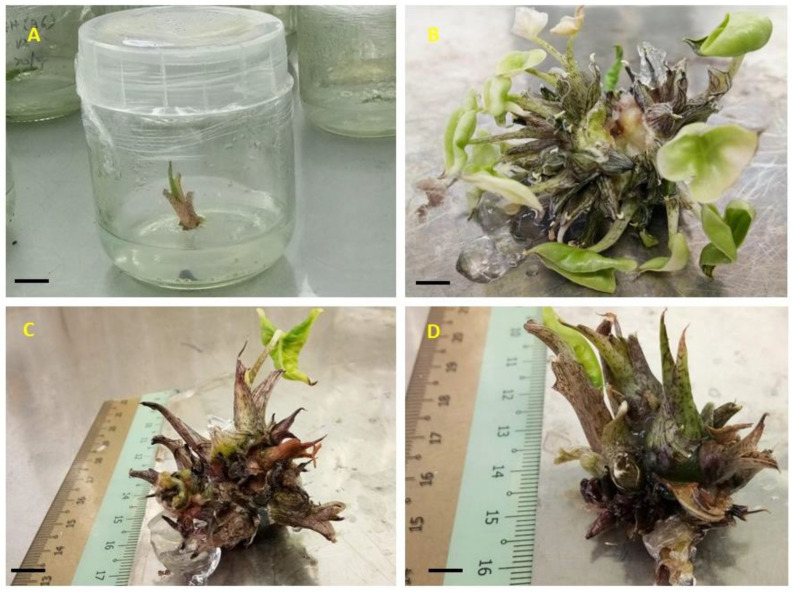
In vitro shoot multiplication from at different subculture stages. (**A**) Individual shoot used for shoot induction and multiplication; (**B**) and (**C**) maximum number of shoots per explant obtained with 3-mg L^−1^ BAP after four weeks; (**D**) highest shoot length obtained with 3-mg L^−1^ TDZ. Bar = 1 cm.

Multiple shoots were induced using different concentration and type of cytokinins alone and in combination with auxins hormones. The percentage of shoot proliferation under different concentrations of BAP was found in the range of 83% to 100%, whereas in KN the shoot proliferation was found in the range of 75–100% meanwhile the shoot proliferation percentage in TDZ was 100% (Table 3). The average shoot number produced using 3-mg L^−1^ BAP was the highest (18.33 ± 2.33) followed by (13.75 ± 2.14) shoots per explant recorded in 4-mg L^−1^ BAP and the lowest (3.66 ± 0.49, 4.50 ± 0.58 and 7.91 ± 1.32) shoots per explant was observed in 1-mg L^−1^, 2-mg L^−1^ and 5-mg L^−1^ BAP, respectively (Figure 4). The highest (12.33 ± 1.02) average shoots per explant in KN treatment was observed in 3-mg L^−1^ KN concentration followed by 4-mg L^−1^ KN which recorded (11.75 ± 1.06) shoots per explant. However, there was no significant difference in average shoots per explant between two treatments. Meanwhile, the lowest average shoots per explants (3.66 ± 0.14) was observed in 1-mg L^−1^ KN.

The shoot length in different concentrations of BAP, KN and TDZ is shown in Table 4. The highest shoot length (7.26 ± 0.56 cm) was recorded in 3-mg L^−1^ TDZ while the lowest (2.44 ± 0.31 cm) average shoot length was observed in 5-mg L^−1^ BAP. The shoot length had decreased significantly as the concentration of BAP, KN and TDZ hormones increased. While the highest shoot length (3.96 ± 1.55 cm) in KN treatment was recorded in 3-mg L^−1^ concentration and the lowest shoot length (2.80 ± 1.01 cm) was observed in 5-mg L^−1^. The effectiveness order of different cytokinins was found to be BAP > TDZ > KN. In the present study, TDZ hormone induced not only shoots, but also produced large number of roots as compared with other cytokinins hormones. Likewise, [43] reported that TDZ hormone at lower concentration could induce shoot proliferation than many other cytokinins, but higher level could inhibit shoot proliferation. However, in the current study the number of shots induced by BAP at 3-mg L^−1^ was the highest.

Our result is in agreement with Arvind et al. 2013, who reported that lower concentration of BAP produced significant highest number of shoots in five *Alocasia* species. The combination of 3-mg L^−1^ and 1.0-mg L^−1^ IAA was most effective for shoot elongation and inducing shoot having roots from shoot tips of *A. longiloba.* In this combination, the highest shoot number per explant was 10.79 ± 1.12 obtained with 3-mg L^−1^ BAP with 1-mg L^−1^ IAA, whereas the lowest shoot number per plant was 5.41 ± 0.58 obtained with 3-mg L^−1^ BAP with 3-mg L^−1^ IAA. In this study, high ratio of both BAP and IAA in the culture medium induced callus. Whereas the low ratios of IAA to BAP induced roots. Our results are in line with [44], who reported that high concentrations of auxin and cytokinins in the culture medium can induces callus, demonstrating high plasticity of plant cells in differentiation and organogenesis. Overall, the current study suggested that 3-mg L^−1^ of BAP is the optimal concentration that can be used to induce large number shoots from shoots tips of *A. longiloba.*

#### 4.1.5. Influence of Auxins on In Vitro Callus Induction

**Figure 5:**
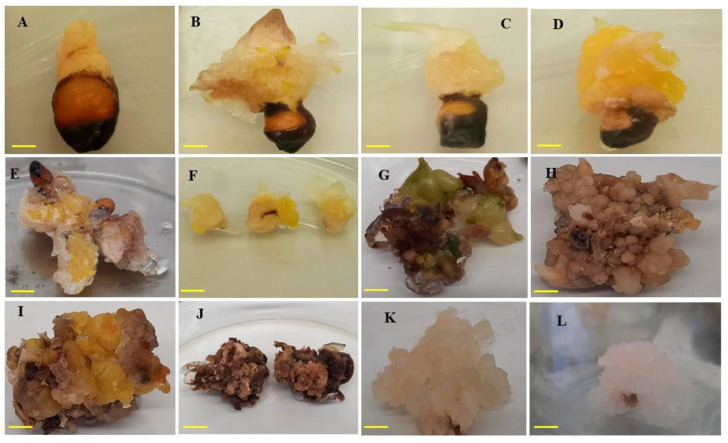
Callus formation on MS media fortified with IAA and PIC alone or in combination with BAP. (**A**) Compact callus induced with 1-mg L^−1^ IAA; (**B**) yellowish compact callus induced with 2-mg L^−1^ IAA; (**C**) and (**D**) yellowish friable callus induced with 3-mg L^−1^ IAA; (**E**) brownish-yellow callus friable texture induced with 3-mg L^−1^ PIC; (**F**) callus obtained from 3-mg L^−1^ IAA and further used for callus proliferation; (**G**–**J**) brown callus from second subculture using combination of IAA with BAP; (**K**–**L**) friable callus from second subculture. Bar = 1 cm.

In vitro callus induction was studied from seeds of *A. longiloba* using different type and concentrations of auxins. In this study, callus induction occurred in approximately 60–80% of seed explants depending on auxin type and concentration. Among different auxins tested, IAA resulted highest percentage of callus induction and FW of callus (Table 4). The IAA treatment at 3-mg L^−1^ exhibited the highest callus induction percentage of 91.66 ± 5.27, followed by 87.50% ± 8.53% in treatment 2.5-mg L^−1^. Meanwhile the lowest callus induction (47.22% ± 9.54%) was recorded in treatment 1-mg L^−1^ IAA. The increase in callus induction % was directly proportional to the increase in IAA concentrations. FW of callus was influenced by the concentration of the growth regulators. The highest FW of callus (6.61 ± 0.58 and 5.24 ± 0.83 g) was obtained with 3-mg L^−1^ and 2-mg L^−1^ IAA, respectively and the lowest FW (3.13 ± 0.42 g) recorded in 1-mg L^−1^ IAA. In the current study, no callus growth was found in control treatment (0-mg IAA or PIC). The callus color observed in this study varies between green, brown and white (Figure 5). The callus induced using treatment IAA were all white and compact except in 2.5-mg L^−1^ and 3-mg L^−1^ concentrations.

Among different concentrations of picloram (PIC) treatment, 3-mg L^−1^ had induced the highest callus induction percentage (84.72% ± 11.05%) followed by 2.5-mg L^−1^ which had recorded 62.50% ± 8.53%. Whereas the lowest callus induction percentage (26.38 ± 6.60) was achieved with 1-mg L^−1^ concentration. The highest FW of callus (6.04 ± 0.82 g) was achieved with 2.5-mg L^−1^ PIC. The callus obtained using different concentrations of PIC were white in color and compact texture.

In the present study, the efficiency of optimal IAA (3-mg L^−1^) combined with different concentrations of BAP were tested to observe if the callus induction percentage and callus weight would be boosted compared to IAA only. The effect of IAA and BAP with various combinations is presented in Table 4. Among different combinations tested, IAA (3-mg L^−1^) along with 0.5-mg L^−1^ BAP was found to be the best combination for high frequency of callus induction with 88.33% ± 5.27%. Maximum callus FW were observed in callus induced by 3-mg L^−1^ IAA along with 3-mg L^−1^ BAP. However, friable callus was formed only on the media supplemented with 3-mg L^−1^ IAA along with 0.5-mg L^−1^ BAP. In this study, it was observed that the callus percentage and callus induction was inhibited in the absence of auxins hormones (control treatment), indicating the significance of plant growth regulators such as auxins for inducing cell division and callus induction. This was proven by several researchers who reported the importance of exogenous application of plant growth regulators for inducing callus formation [45,46,47].

#### 4.1.6. In Vitro Root Formation and Acclimatization of In Vitro Raised Plantlets

In this study, lower concentration of IAA were found to be effective for root induction and plantlet development. This was demonstrated in terms of higher rooting percentage, number of roots formed per explant and the average length of root formed. Based on the results obtained, the maximum rooting percentage (95%) resulted from the explants in medium supplemented with 0.5-mg L^−1^ IAA. The rooting percentage induced with 0, 1 and 2-mg L^−1^ were 80%, 90% and 85%, respectively (Table 5). The rooting percentage had declined as the concentration of IAA increased. The maximum root number per shoot (11.41 ± 2.16) was achieved in 0.5-mg L^−1^ IAA. The root number per shoot decreases as the concentration of IAA increase. Among different IAA concentrations, the longest average root length (9.61 ± 1.43 cm) was achieved on MS media supplemented with 0.5-mg L^−1^ IAA. While the shortest root-length (6.51 ± 1.08 cm) was recorded in 2-mg L^−1^ IAA. Activated charcoal at 3 g L^−1^ induced (100%) rooting response however the mean number of roots per shoot (5.01 ± 1.59) was found to be lower.

In the current study, the MS medium supplemented with 0.5-mg L^−1^ IAA demonstrated a better response with the highest percentage of rooting as well as the longest root formation than the other concentrations of IAA tested. Meanwhile, as the concentration of IAA increased from 0.5 to 2-mg L^−1^, lower rooting efficiency was observed. This could be due to the fact that high concentrations of auxins could have herbicidal property which impede the adventitious root induction and may have also other effects on plant tissues. The growth abnormality caused by high concentration results from endogenous hormonal imbalance [48]. Therefore, the use of appropriate concentration of auxin are important to control the growth and development of plants in vitro. This is confirmed by the result of research on *Daphne mezereum* L. ‘Alba’ where they reported optimum rooting formation using lower concentration of auxin [49]. The present study suggested that IAA at the concentration of 0.5-mg L^−1^ is the optimum to induce root.

**Figure 6:**
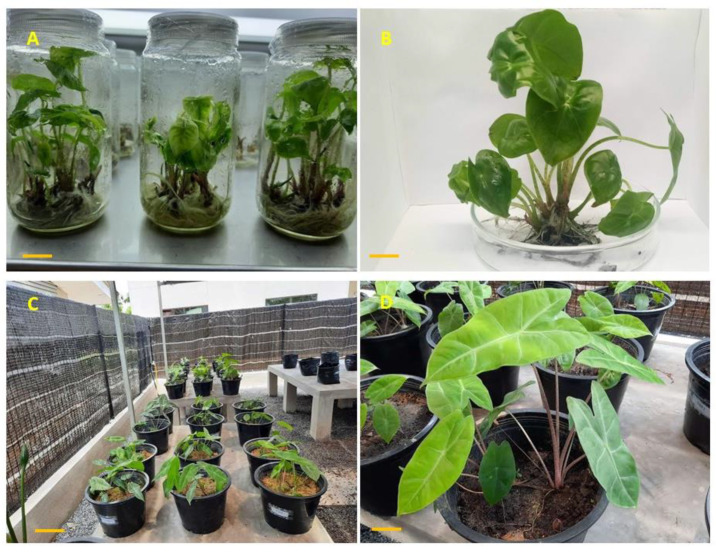
*A. longiloba* plantlets obtained from in vitro culture and acclimatization process; (**A**) In vitro plantlets ready to be acclimatized; (**B**) plantlets taken out from culture vessel about 9–10 cm tall; (**C**) acclimatized plant in the green house where there is 100% shade; (**D**) *A. longiloba* plant after 45 d of acclimatization.

All the in vitro plantlets (Figure 6A) obtained from earlier experiments (in vitro rooting) were transferred to the greenhouse for hardening process (Figure 6C). After six weeks of acclimatization, the plantlets showed vigorous growth with the survival rate of 97%. The highest average petiole height (12.33 ± 1.01 cm) was observed at T4 (combination of topsoil and peat moss at 1:2 ratio) followed by T3, T5 and T6, respectively (Table 6). The largest average leaf length (16.75 ± 1.64 cm) was observed in T4, however it was not found statistically significant different with T1, T3 and T5 which recorded 13.79 ± 0.94 cm, 14.66 ± 0.91 cm and 15.21 ± 0.98 cm, respectively. The shortest average leaf length (9.99 ± 1.14 cm) were recorded in T2. The maximum average leaf width (6.19 ± 0.35 cm) was recorded in T4 and the shortest leaf width (4.86 ± 0.22 cm) observed in T6. Significant higher number of leaves per plant (9.16 ± 1.88) was observed in T2. Overall, among different soil media combination, T4 was found to be the best soil media for the acclimatization of *A. longiloba* plantlets (Figure 6D) and moreover the plantlets used in this experiment responded optimally when acclimatized in combination than a single soil media. This could be due the presence of essential soil nutrients in the topsoil and high concentration decomposed organic matter mostly made from dead biologic matters such as microorganism, dead insect, plants and so forth. Likewise, peat moss substrate contains sphagnum moss and organic matter that promotes faster growth. As reported by [50], the potting media rich in nutrient content is essential for vegetative growth and minimize the time taken to maturity and flower formation. Our finding are in line with [51] who reported maximum plant growth in organic soil and peat moss substrates.

### 4.2. Total Phenolic and Flavonoid Content

Comparative study were carried out to determine the total phenolics (TPC) and flavonoids content (TFC) of field-grown plant parts (fruit and petiole), in vitro-derived greenhouse-grown plant part (petiole) and in vitro derived callus. In this study, the TPC of extracts of the in vitro-derived callus was 538 mg GA equivalent/g, whereas the field-grown plant parts (fruit and petiole) and in vitro-derived greenhouse-grown plant part (petiole) showed 504, 300 and 98 mg GA equivalent/g, respectively (Table 7). The TPC of in vitro-derived callus is significantly (*p* < 0.05) high than all extracts tested. This in agreement with the results of [6], whereby higher TPC content was found in the holy basil’s callus extracts compared with other field-grown plant parts. [3], explained that the culture medium used to grow a callus tissue is having more carbon influx that may influence the biosynthesis of elevated levels of phenols. Therefore, in vitro-derived callus can be an alternative source of desired bioactive compounds without destroying the wild plant resources. Further in this study, the in vitro regenerated plants exhibited lower TFC compared to field-grown plant parts. This property could be due to several factors that may affects secondary metabolite production such as the age of the plant, environmental factors, time of collection and so forth. Other studies have reported that environmental factors such as light intensity and carbon dioxide concentration affects concentration of secondary metabolites [52]. In addition, phytochemicals such as phenols, flavonoids and alkaloids, to some extent under genetic control, but they are also significantly affected by changing growth conditions rising from climate and soil condition [53].

The total flavonoids content in the ethanolic extract of petiole, fruit, in vitro-derived greenhouse-grown plant and in vitro-derived callus was found to be 449, 441, 114 and 236 mg QCE /g. In this study, the highest TFC was observed in the field grown plant part (petiole), however there was no significance difference with that of fruit extracts. The TFC content in callus extract was relatively lower. However, the in vitro-derived greenhouse-grown plant had lowest total flavonoids content as compared with all plant parts.

### 4.3. Effect of Plant Extracts on DPPH and ABTS Radical Scavenging Activity

The antioxidant activity of herbal extracts was shown to be involved in the quenching of free radical reactions. DPPH and ABTS molecules which contains stable free radicals was used to evaluate the radical scavenging activity of plant extracts. Scavenging activity (anti-radical) capacity of plant extracts can be assessed by measuring decrease in the absorbance of DPPH and ABTS at 517 nm and 734 nm, respectively. In this study, we evaluated antioxidant properties of field-grown plant parts, i.e., petiole and fruit extracts, in vitro-derived greenhouse-grown plant and in vitro-*derived* callus. Results of the current study showed that all *A. longiloba* field-grown plant part (fruit and petiole), in vitro-derived callus and in vitro-derived greenhouse-grown plant extracts were able to scavenge DPPH and ABTS radicals in a concentration dependent manner. The in vitro-derived callus exhibited better DPPH radical scavenging activity than field-grown plant parts, i.e., petiole and fruit extracts and in vitro-derived greenhouse-grown plant. In this study, EC_50_ value of the in vitro-derived callus was 0.113 ± 0.120-mg mL^−1^, whereas the extracts of petiole, fruit and in vitro plant showed 0.126 ± 0.058-, 0.137- ± 0.005- and 0.173- ± 0.006-mg mL^−1^, respectively (Table 8). The EC_50_ of in vitro-derived callus extracts required to quench 50% DPPH radicals is significantly (*p* < 0.05) less than the extract of field-grown and in vitro-derived greenhouse-grown plant parts. This property could be due to high accumulation of phenolic compounds in callus extracts. The present study revealed that the extracts of in vitro-derived callus and field-grown plant parts act as a free radical inhibitor and thus are attributed as primary antioxidants that react with free radicals. Further in this study, high linear correlation was obtained between TPC and DPPH radical scavenging activity in all extracts. These results shown that radical scavenging activity of *A. longiloba* extracts could be mostly related to their TPC. The anti-free-radicals property of phenolic compounds depends on their molecular structures, particularly the availability of phenolic hydrogens leads to the formation of phenoxyl radicals by hydrogen donation and confers to phenolic compounds the ability of in-activating free radicals [54,55,56]. Using the DPPH assay, the antioxidant activities of *A. longiloba* field-grown plant parts and in vitro-derived callus extract were ranked in the order in vitro-derived callus > petiole > fruit. Therefore, the TPC in each extract may be responsible for the antioxidant properties studied.

The ABTS free radical scavenging activity of *A. longiloba* field-grown plant parts (fruit and petiole), in vitro-derived callus and in vitro-derived greenhouse-grown plant extracts were studied. The fruit extracts showed better scavenging activity than all extracts tested (Table 8). In this study, the EC_50_ value of the extracts of fruit was 0.088 ± 0.017-mg mL^−1^, whereas the extracts of petiole, callus and in vitro plant showed 0.088 ± 0.017, 0.108 ± 0.058 and 0.139 ± 0.059-mg mL^−1^, respectively. The EC_50_ required to scavenge 50% ABTS radicals is significantly (*p* < 0.005) less than all the extracts used in the experiment. The present study revealed that the extracts of *A. longiloba* field-grown plant parts and in vitro-derived callus act as free radical inhibitors and thus are attributed as primary antioxidants that react with free radicals.

## 5. Conclusions

The present study demonstrates an efficient micropropagation protocols for *A. longiloba,* as well as comparative study on the antioxidant properties of ethanolic extracts of the field-grown plant, in vitro-derived greenhouse-grown plant and in vitro-derived callus for the first time. In this study, the plant growth regulators that can induce high shoot multiplication, high rooting and survival percentages were identified. Further in this study, a comparison of antioxidant properties of ethanolic extracts of the field-grown plant, in vitro-derived greenhouse-grown plant and in vitro derived callus shows that in vitro-derived callus could be favored for high TPC and better DPPH radical scavenging activity. The fruit extracts also showed excellent ABTS radical scavenging activity as well as high TFC. Therefore, both micropropagation and callus culture techniques offers an opportunity to produce large number of plants and improve the secondary metabolites production in terms of quality and quantity. Overall, the current study suggests that the micropropagation protocol established in the present study could help in rapid and mass multiplication of *A. longiloba* plant and the callus extracts could be used for their high antioxidant activity.

## Figures and Tables

**Table 1:** Mean contamination level (%) and mean survival rate (%) of *A. longiloba* seeds one month after exposure to different sterilization treatments.

Sterilization Treatment (Clorox)	Contamination (%)	Survival (%)
T1 (10%)	62.50 ± 6.15 ^c^	32.50 ± 6.80 ^c^
T2 (20%)	37.50 ± 10.22 ^b^	55.00 ± 9.83 ^b^
T3 (30%)	16.66 ± 8.02 ^a,b^	79.66 ± 7.57 ^a^
T4 (40%)	5.83 ± 2.71 ^a^	78.66 ± 4.17 ^a^

* Means followed by the same letter(s) within each column are not significantly different (*p* < 0.05) using DMRT. n = 6. Letters (i.e., a, b, c, d) were used to indicate statistical differences between means.

**Table 2:** Breaking seed dormancy of *A. longiloba* seeds by sulfuric acid treatment with seed coat breaking.

Pre-Germination Treatments		Germination (%)	Number of Days to Germinate
Sulfuric Acid (%)	Gibberellic Acid (mg L^−1^)		
Control (MSO)	-	25.00 ± 6.45 ^d^	30.16 ± 1.57 ^b^
10	-	45.83 ± 7.68 ^b^	25.00 ± 2.94 ^a,b^
20	-	79.16 ± 7.68 ^a,b^	20.50 ± 3.06 ^a^
30	-	87.50 ± 5.59 ^a^	19.00 ± 1.15 ^a^
40	-	62.50 ± 5.59 ^b,c^	20.66 ± 3.01 ^a^
	Control (DW)	24.16 ± 6.11 ^b^	31.33 ± 2.66 ^b^
	5	25.00 ± 5.00 ^b^	28.33 ± 1.58 ^a,b^
	10	27.50 ± 4.03 ^b^	27.66 ± 2.02 ^a,b^
	15	46.66 ± 9.88 ^a,b^	24.66 ± 2.36 ^a,b^
	20	53.33 ± 9.88 ^a^	22.83 ± 3.77 ^a^

* Means followed by the same letter (s) within each column are not significantly different (*p* < 0.05) using DMRT. n = 6. Letters (i.e., a, b, c, d) were used to indicate statistical differences between means.

**Table 3:** Influence of cytokinins on multiple shoot bud induction of *A. longiloba* using an individual shoots.

Growth Regulators (mg L^−1^)				Shoot Response (%)	Number of Shoots/Explant	Shoot Length (cm)
BAP	KN	TDZ	IAA			
1.0	–			83.33	3.66 ± 0.49 ^c,d^	3.87 ± 0.36 ^a^
2.0	–			100.0	4.50 ± 0.58 ^c,d^	3.21 ± 0.28 ^a,b^
3.0	–			100.0	18.33 ± 2.33 ^a^	3.05 ± 0.44 ^a,b^
4.0	–			100.0	13.75 ± 2.14 ^b^	2.82 ± 0.28 ^b^
5.0	–			100.0	7.91 ± 1.32 ^c^	2.44 ± 0.31 ^b^
	1.0	–		75.00	3.66 ± 0.14 ^c^	3.35 ± 0.78 ^a,b^
	2.0	–		100.0	4.20 ± 0.55 ^c^	3.72 ± 1.43 ^a,b^
	3.0	–		100.0	12.33 ± 1.02 ^a^	3.96 ± 1.55 ^a^
	4.0	–		100.0	11.75 ± 1.06 ^a^	3.23 ± 0.66 ^a,b^
	5.0	–		100.0	8.00 ± 0.93 ^b^	2.80 ± 1.01 ^b^
		1.0	–	100.0	5.08 ± 0.76 ^c^	5.91 ± 0.36 ^a,b^
		2.0	–	100.0	6.25 ± 0.66 ^b,c^	6.38 ± 0.76 ^a,b^
		3.0	–	100.0	11.83 ± 1.35 ^a^	7.26 ± 0.56 ^a^
		4.0	–	100.0	8.75 ± 1.16 ^b^	5.38 ± 0.48 ^b^
		5.0	–	100.0	7.41 ± 0.78b ^c^	5.25 ± 0.36 ^b^
3.0			0.5	100.0	10.45 ± 1.10 ^a^	6.78 ± 0.73 ^a^
3.0			1.0	100.0	10.79 ± 1.12 ^a^	6.22 ± 0.58 ^a^
3.0			1.5	100.0	8.37 ± 1.12 ^a,b^	6.11 ± 0.29 ^a^
3.0			2.0	100.0	8.08 ± 0.97 ^b,c^	5.69 ± 0.50 ^a^
3.0			2.5	100.0	5.83 ± 0.34 ^b,c^	4.26 ± 0.29 ^b^
3.0			3.0	100.0	5.41 ± 0.58 ^c^	4.11 ± 0.27 ^b^

* Means followed by the same letter (s) within each column are not significantly different (*p* < 0.05) using DMRT. Letters (i.e., a, b, c, d) were used to indicate statistical differences between means.

**Table 4:** Influence of auxins alone and in combination with cytokinins on callus induction from *Alocasia longiloba* seed.

Growth Regulators (mg L^−1^)			% Callus Induction	Fresh Weight (FW) (g)	Color	Texture
IAA	PIC	BAP				
0.0	–		0.00 ± 00	0.00 ± 00	-	-
1.0	–		47.22 ± 9.54 ^c^	3.13 ± 0.42 ^b^	White	Compact
1.5	–		54.16 ± 7.68 ^c^	4.98 ± 0.50 ^a,b^	White	Compact
2.0	–		62.50 ± 5.59 ^b,c^	5.24 ± 0.83 ^a,b^	Yellow/brown	Compact
2.5	–		87.50 ± 8.53 ^a,b^	4.19 ± 0.65 ^a,b^	White	Friable
3.0	–		91.66 ± 5.27 ^a^	6.61 ± 0.58 ^a^	Yellow	Friable
	1.0	–	26.38 ± 6.60 ^c^	2.27 ± 0.46 ^c^	White	Compact
	1.5	–	34.72 ± 8.16 ^b,c^	3.28 ± 0.24 ^b,c^	White	Compact
	2.0	–	51.38 ± 5.45 ^b,c^	4.91 ± 0.80 ^a,b^	White	Compact
	2.5	–	62.50 ± 8.53 ^a,b^	6.04 ± 0.82 ^a^	White	Compact
	3.0	–	84.72 ± 11.05 ^a^	5.18 ± 0.72 ^a,b^	Yellow/brown	friable
3.0		0.5	88.33 ± 5.27 ^a^	3.20 ± 0.59 ^a^	Creamy	Friable
3.0		1.0	83.33 ± 8.33 ^a,b^	3.93 ± 0.62 ^a^	Cream white	Nodular
3.0		1.5	79.16 ± 7.68 ^a,b^	4.05 ± 0.59 ^a^	Green	Nodular
3.0		2.0	70. 83 ± 10.03 ^a,b^	4.50 ± 0.62 ^a^	Brown	Nodular
3.0		3.0	58.33 ± 10.54 ^b^	4.56 ± 0.40 ^a^	Green	Nodular

* Means followed by the same letter are not statistically significant at (*p* < 0.05). n = 12. Letters (i.e., a, b, c, d) were used to indicate statistical differences between means.

**Table 5:** Effect of auxins on in vitro root induction in micro-propagated shoots after four weeks of culture.

Growth Regulators		% Shoot Rooted	Mean Root No/Shoot	Mean Root Length (cm)
IAA (mg L^−1^)	AC (g L^−1^)			
0.0	-	80.0	6.91 ± 0.63 ^b^	9.30 ± 1.18 ^a^
0.5	-	95.0	11.41 ± 2.16 ^a^	9.61 ± 1.43 ^a^
1.0	-	90.0	8.83 ± 1.11 ^a,b^	7.53 ± 1.02 ^a,b^
2.0	-	85.0	8.58 ± 1.51 ^a,b^	6.51 ± 1.08 ^b^
	1.0	100.0	4.66 ± 0.71 ^b^	6.01 ± 2.04 ^a^
	1.5	100.0	8.41 ± 1.24 ^a^	5.58 ± 1.41 ^a^
	2.0	100.0	10.58 ± 1.89 ^a^	5.28 ± 0.67 ^a^
	3.0	100.0	12.16 ± 0.66 ^a^	5.01 ± 1.59 ^a^

* Means with the same letter within the same column are not significantly different at *p* < 0.05 according to Duncan’s multiple range test (DMRT), n = 12. Letters (i.e., a, b, c, d) were used to indicate statistical differences between means.

**Table 6:** Effect of different composition of soil media on the growth of plantlets in the greenhouse.

Soil Composition (Treatments)	Growth Parameters			
	Petiole Length (cm)	Leaf Length (cm)	Leaf Width (cm)	Leaf Number
T1 (TS)	10.19 ± 0.81 ^a,b^	13.79 ± 0.94 ^a,b^	5.20 ± 0.31 ^a,b^	5.30 ± 0.58 ^b^
T2 (PM)	7.61 ± 0.93 ^b^	9.99 ± 1.14 ^c^	4.40 ± 0.44 ^b^	9.16 ± 1.88 ^a^
T3 (TS:PM) 1:1	11.56 ± 0.76 ^a^	14.66 ± 0.91 ^a,b^	5.43 ± 0.44 ^a,b^	5.08 ± 0.45 ^b^
T4 (TS: PM) 1:2	12.33 ± 1.01 ^a^	16.75 ± 1.64 ^a^	6.19 ± 0.35 ^a^	5.91 ± 0.27 ^b^
T5 (TS: PM) 1:3	11.56 ± 1.22 ^a^	15.21 ± 0.98 ^a,b^	6.04 ± 0.27 ^a^	4.83 ± 0.16 ^b^
T6 (PM:SD) 2:1	9.72 ± 1.26 ^a,b^	12.06 ±0.98 ^b,c^	4.86 ± 0.22 ^b^	4.41 ± 0.92 ^b^

* Means followed by the same letter are not statistically different after testing in one-way ANOVA and DMRT *p* < 0.05. n = 12. TS = topsoil; PM = peat moss; SD = sand. Letters (i.e., a, b, c, d) were used to indicate statistical differences between means.

**Table 7:** Total flavonoids and phenolics content of the field-grown plant parts, in vitro propagated and in vitro-derived callus.

Plant	Plant Parts Used	Total Phenolic Content mg GAE/g	Total Flavonoids Content mg QCE /g
Field-grown	Petiole	300.40 ± 7.66 ^c^	449.46 ± 0.78 ^a^
	Fruits	504.44 ± 4.25 ^b^	441.30 ± 3.26 ^a,b^
In vitro raised	Petiole	98.18 ± 0.36 ^d^	114.86 ± 0.33 ^d^
Callus	Friable callus	538.43 ± 0.64 ^a^	236.40 ± 0.61 ^c^

* Means with the same letter within the same column are not significantly different at *p* < 0.05 according to Duncan’s multiple range test (DMRT), n = 3. Letters (i.e., a, b, c, d) were used to indicate statistical differences between means.

**Table 8:** Antioxidant activity of the field-grown plant parts, in vitro propagated and in vitro-derived callus of *A. longiloba.*

Extracts	Plant Parts Used	EC_50_ (mg mL^−1^)	
		DPPH	ABTS
Field-grown	Petiole	0.126 ± 0.058^b^	0.088 ± 0.017 ^b^
	Fruits	0.137 ± 0.005 ^b^	0.083 ± 0.058 ^a^
In vitro raised plants	Petiole	0.173 ± 0.006 ^c^	0.139 ± 0.059 ^d^
Callus	Friable callus	0.113 ± 0.120 ^a^	0.108 ± 0.058 ^c^

* Means with the same letter within the same column are not significantly different at *p* < 0.05 according to Duncan’s multiple range test (DMRT), n = 3. Letters (i.e., a, b, c, d) were used to indicate statistical differences between means.
